# Maximising ecological value and assessing land suitability for sustainable grassland management in Asia’s largest tropical grassland, Western India

**DOI:** 10.1038/s41598-024-62775-9

**Published:** 2024-06-13

**Authors:** Rupak Dey, Seema B. Sharma, Mahesh G. Thakkar

**Affiliations:** 1 Department of Earth and Environmental Science, KSKV Kachchh University, Mundra Road, Bhuj, Kachchh, Gujarat 370001 India; 2 Birbal Sahni Institute of Palaeosciences, Lucknow, India

**Keywords:** Land suitability, Multi criteria evolution, Analytical hierarchy process, Semi arid, Grassland, Climate sciences, Ecology, Environmental sciences

## Abstract

Grasslands are crucial ecosystems that provide numerous ecological services and support biodiversity conservation. Grasslands undergo significant threats from both anthropogenic and natural sources, compromising their ability to maintain biodiversity, ecosystem services, and human well-being. However, grasslands are frequently ignored in sustainable development objectives. Adequate knowledge of how grassland degradation affects ecosystem services is essential for sustainable management and grassland ecological restoration. The Kachchh region in western India harbours a unique grassland ecosystem known as the Banni grassland, which once became the finest grassland in Asia. However, undesirable anthropogenic interventions have accelerated its degradation. This research paper aims to assess the suitability of different land areas in Banni for sustainable grassland restoration, considering ecological value as a primary criterion. In the present research, land suitability for grassland management was assessed using a geographical information system (GIS)-based multi criteria evolution (MCE) method with satellite data and the analytic hierarchy process (AHP). The ground truthing of the soil samples was carried out alongside. Slope, rainfall, infiltration rate, LULC, geomorphology, soil texture, soil organic carbon, water holding capacity, SAR, CEC, pH, EC, and soil nutrients were among the criteria used. The weights for each criterion were calculated using a pairwise comparison matrix, and the scores were allocated to sub criteria based on field work, expert opinions, and a literature review. The proposed method can be very useful for evaluating the state of the land and can help with the best possible planning for grassland development and conservation. Banni grassland has the potential to be developed into a critical zone observatory (CZO) in the future, and the present study, with further inputs, holds promise for furthering the cause of its sustainable management. Overall, this study underscores the importance of assessing land suitability for sustainable grassland management and highlights the potential for maximising the ecological value of grasslands in western India and beyond.

## Introduction

Grasslands, which include savannas, grassy shrublands, and open grasslands, make up 30–40% of the earth’s surface; they are distributed mainly in semiarid and arid areas and are an important component of terrestrial ecosystems, and their degradation is a serious ecological problem that has a negative impact on ecosystem services and constitutes a serious threat to the health of grassland ecosystems^1–5^.The vast majority of iconic and unique species may be found in grasslands, which also offer a variety of material and intangible advantages to people^6^. Grasslands are an essential worldwide biodiversity repository. These advantages include several ecosystem services, including those related to food production, water supply and management, carbon storage and climate mitigation, pollination, and a plethora of cultural services^7–9^. Global desertification and degradation of grasslands have been caused by deforestation, overgrazing, agriculture, the ejection of native species, and urbanisation accompanied by population increases, putting the services and functions of these ecosystems at risk. The carbon cycle, local economy, climate, and rates of grassland reproduction and germination might all be significantly impacted by grassland degradation^10^.Despite the importance of grasslands, they are being degraded rapidly and widely in many regions of the world, with as much as 49% of grassland areas worldwide experiencing a certain level of degradation^11,12^. In India, grasslands makes approximately 788,943 sq. km, or approximately 24% of the country’s total land area (32,87,263 sq. km)^13^ and are increasingly threatened by agricultural conversion, tree-based plantation projects, invasive species, fire suppression, and mega-development projects^14^. Indian grasslands are negatively affected by India’s commitment to global goals and the involvement of multiple government bodies in grassland management. This issue is exacerbated by the strong forest bias of government and non-profit organisations. Lahiri et al.^14^ proposed that for India to effectively preserve and regenerate grasslands, a more unified national policy framework and a strong ecological categorisation system are needed. The Kachchh district, which is situated in Gujarat, is characterised by having vast stretches of saline desert, salt marshes and grasslands. The grasslands in this district are popularly known as ‘Banni’, which once covered an area of approximately 3800 sq. km^15^ but decreased to 2618 sq. km^13^.The region possessed an innate salinity from its early days, but the rivers (Khari, Bhurud, Nara, Kalia, Kaswati, and Panjora) that flowed from the Kachchh mainland to Banni were not only depositing debris but also leaching the salinity during good rainfall years. Salinity was therefore not a major issue in the past. Rudramata, Nirona, Nara, Kaila, Kaswati, and Gajansar are six medium-sized dams that were built along rivers approximately 1960 and completely blocked the flow of fresh water into Banni. They also restricted the delivery of nutrients and the leaching of salinity, except for years with exceptionally strong rainfall^16^. Although several factors contribute to the degradation of the Banni grassland, it is still unclear whether the invasion of Prosopis juliflora or an increase in salinity is the primary cause^16^. One of India’s least protected, most misused, and most ignored ecosystems is grasslands; unless they are designated as protected areas by the Wild Life (Protection) Act of 1972 or as reserve forests by the Indian Forest Act of 1927, they are not protected^17^. Banni was declared a protected forest under Sect.  29 of the Indian forest act, 1927, via chief commissioner of Kachchh’s Notification No. RR/155/55 dated 11-5-1955, but it was not transferred to the forest department for scientific management and has remained in the revenue department’s possession since then. The whole Banni protected forest was subsequently designated a “Conservation Reserve” within the Kachchh Desert Wildlife Sanctuary, which includes the Chhari dhandh. As a result, in 2009, a working plan for an area of 1887 sq. km was prepared (Working Plan of Banni Protected Forests 2009), which grouped Banni into three major circles, and the best part was to classify this land as grassland rather than forestland. In light of this, several governmental and nongovernmental groups have carried out studies and proposed corrective actions to enhance Banni’s general range condition^18–23^. However, the majority of such recommendations were either never implemented or implemented without an ecological perspective^16^. As a result, restoring Banni and returning it to a productive and sustainable environment necessitate a comprehensive ecological strategy.

The development of grassland conservation zones is essential for avoiding new disturbances and restoring degraded grasslands^24^. The majority of research on grassland degradation has been performed at the sample plot scale, but studies at the regional scale frequently use single vegetation indicators, such as net primary productivity (NPP) and the normalised difference vegetation index (NDVI), to assess grassland degradation without accounting for soil characteristics, which could compromise the accuracy of suitability analysis and grassland degradation assessment^25,26^. Land decision-makers must choose the best framework for determining whether a piece of land is suitable for grassland protection. The type and quantity of information available, extent of the land surface, laws, and potential for future data collection all have a significant impact on these zoning decisions. The development and implementation of the zoning scheme is perhaps the step that has the most bearing on the design of ecological protection zones. The units of the area to be preserved are given specified purposes through zoning. Land zoning has been significantly impacted by the availability of spatial tools and computational technology to aid in decision-making^27–30^ through improved land management, reduced land degradation, and the establishment of land use patterns that prevent environmental problems by separating conflicting land uses^31,32^. Planning for cropland and grassland land use begins with a land suitability evaluation, which is typically performed to determine whether a certain land use is appropriate for a given location^28^. A technique for evaluating a piece of land called land suitability assessment identifies the main suitability zones for grassland restoration^28,33^. Identifying the suitability and quality of land is important for deciding on land use depending on its potential and safeguarding natural resources for future generations^34^. Effective grassland management requires a comprehensive understanding of land suitability, which involves assessing the compatibility of land for specific uses. Land suitability analysis aids in identifying suitable areas for grassland restoration, optimising livestock forage production, and facilitating sustainable land management practices. In general, land assessment approaches are classified as either qualitative (based on expert knowledge) or quantitative (based on simulation models)^35^. Quantitative models are very sophisticated for land performance, and they frequently require a large amount of data, effort, and money. Land and soil properties, on the other hand, are articulated mathematically in qualitative techniques to determine land suitability. In this scenario, assessing land suitability for land operations is understandably considered a challenging topic with various factors. In other words, a multi criteria assessment technique would be more suited for land evaluation analysis investigations. These issues may now be solved by applying tools such as Multi-criteria decision analysis (MCDA) to perform reasonable assessments and evaluations, in addition to current techniques such as remote sensing and geographic information systems (GIS)^28,29,36–40^. The analytic hierarchy process^41^ (AHP), which provides weights to assessment criteria, is one of the most often utilised MCDA approaches. AHP is capable of detecting and integrating discrepancies in decision-making^42^. A priority vector is often computed based on a pairwise comparison arising from a value chosen by experts on a 1–9 scale.

The goal of this study was to identify potential areas suitable for grassland restoration in the Banni grassland, which has a semiarid terrestrial ecosystem, using the MCDA technique in the analytic hierarchy process (AHP) environment. Furthermore, the primary hypothesis of this study is aimed not only at assisting in the identification of lands suitable for grassland development but also at assisting in the sustainable management of territories by assessing the characteristics of soils, which are the most significant and vulnerable components of arid and semiarid terrestrial ecosystems.

## Materials and methods

### Study area

**Figure 1:**
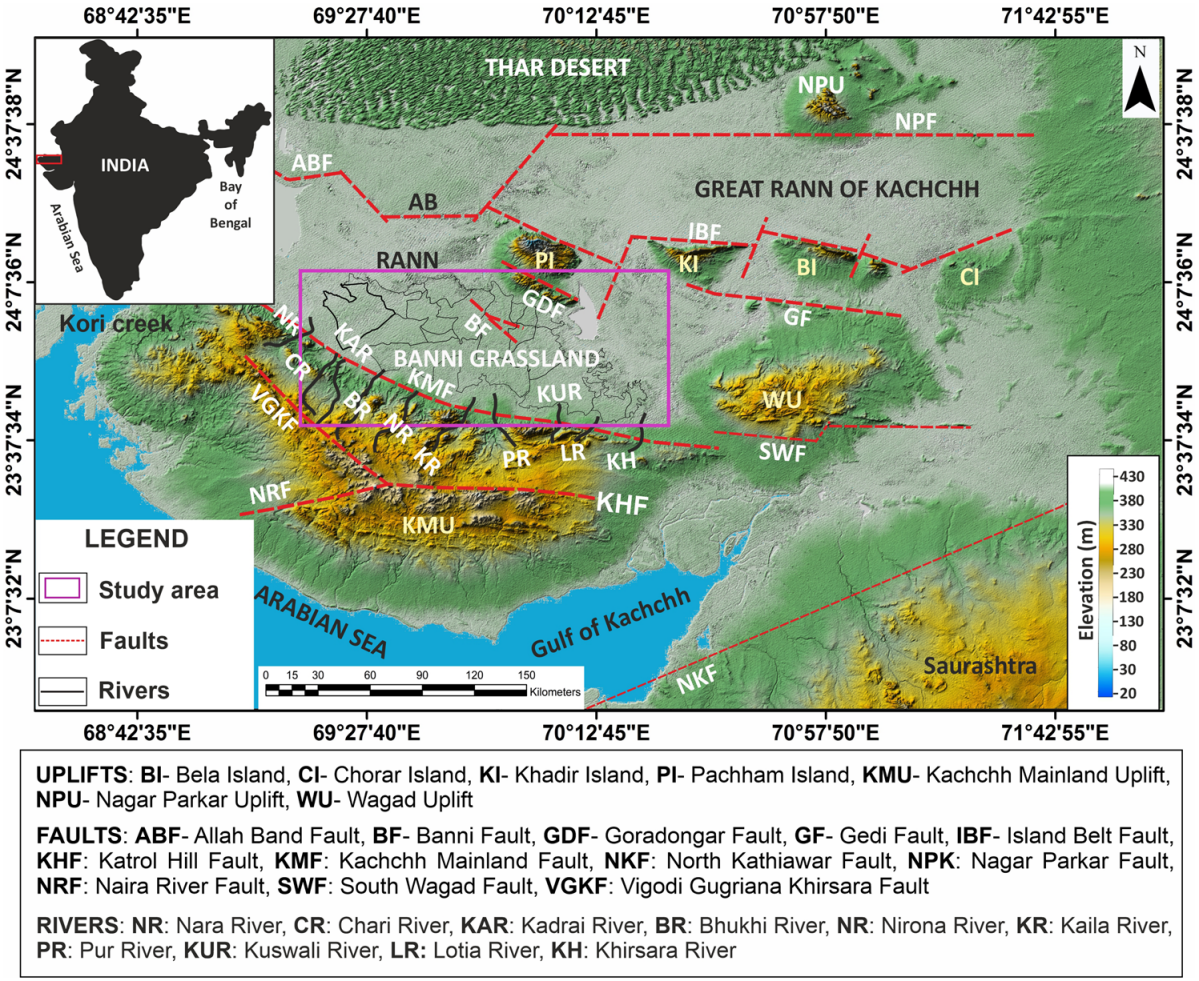
Morphotectonic map of the study area showing major north flowing ephemeral streams into the Banni. The map prepared by authors using software Global mapper version 24.1 (https://www.bluemarblegeo.com/global-mapper-download/) and Corel draw version ×8 (https://www.coreldraw.com/en/pages/coreldraw-x8/).

The Banni plains are among the most enigmatic geological features in the Kachchh region, Western India (23° 19′ to 23^°^ 52′ N latitude and 68° 56′ to 70^°^ 32′ E longitude). It is a highly semiarid fragile ecosystem with slightly undulating and rough terrain. Banni plains are predominantly flat saline land with several shallow depressions, which act as seasonal wetlands after monsoons, and during winters, they convert into sedge mixed grasslands, an ideal dual ecosystem. The yearly rainfall in the area is unpredictable and low (mean 288 mm), with a coefficient of variation ranging from 60 to 80%. The average annual rainfall is approximately 317 mm^13,43^. The monsoon season, which lasts from June to September, brings more than 80% of the annual precipitation. The average temperature of Banni varies from 49 °C in the summer (May-June) to 10 °C in the winter (January-February)^13^. Banni is dominated by low-growing forbs and graminoids, which are mostly halophiles (salt tolerant), as well as scattered tree cover and scrub. The tree cover consists of Salvadora spp. and the invasive Prosopisjuliflora while Cressacretica, Cyperus spp., Sporobolus, Dichanthium, and Aristida are the dominant species found in the area^44^. The soils of Banni are saline sodic, posing a threat to cultivation of any crop except the halophytic grasses and sturdy plant species^43^. The soils belong majorly to cambic arenosols in high level mudflats, whereas in low level mudflats solanchaks are dominant^43^. Geologically, the plains of Banni are tectonically raised mudflats of the earlier Rann surface, with a vast flat terrain. Itis highly vegetated with thorny shrubs and grasses and extends from mainland Kachchh in the south to Pachham Island and the Great Rann in the north^43,45^. It is a distinctive geomorphic surface of the Great Rann that is located at the greatest altitude and is, as a result, entirely devoid of current marine influence. Geologically, the Banni Plains are the deposition sites for Late Pleistocene to Holocene sediments from the northern river systems, rivers of various uplifts of the Kachchh basin and marine sediments of the Arabian Sea from the west^46–48^. The geomorphology reveals that the Banni Plains are a large palaeo mud flat with an elevation of 2–12 m above mean sea level. There are many ephemeral streams, viz. Nara, Chari, Kadrai, Bhukhi, Nirona, Kaila, Pur, Kasvali, Lotia and Khirsara flow across the Kachchh mainland fault (KMF) and into the Banni Plains from the south (Fig. 1). These ephemeral rivers deposited alluvial fan sediments, which cover the southern end of the Banni Plains. The Banni Plains are constrained to the south by the Kachchh mainland fault and to the north by the Banni fault^49,50^.

### Experimental design and data collection

**Figure 2:**
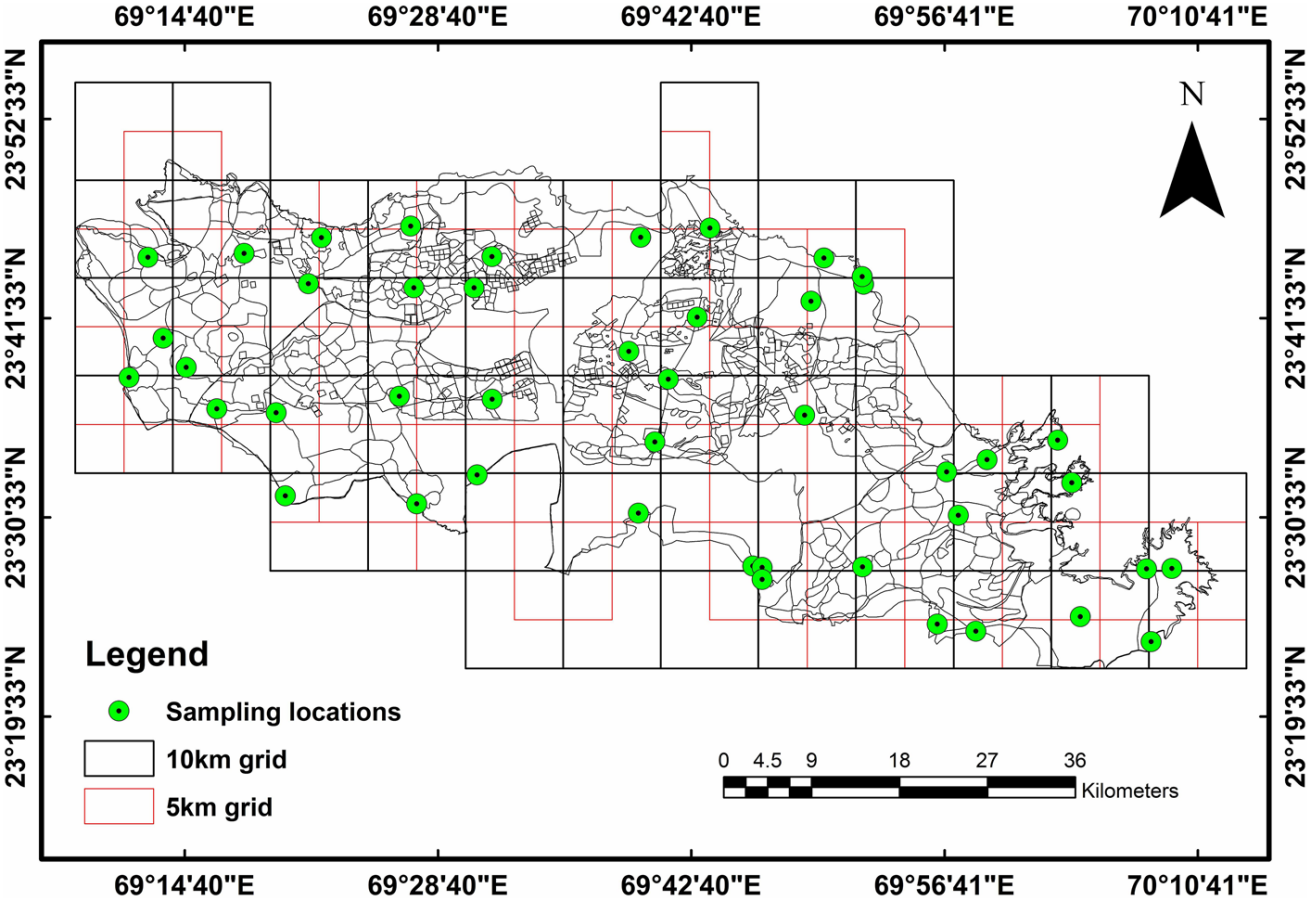
Soil sampling strategy. The map prepared by authors using Arc GIS version 10.7 (https://desktop.arcgis.com/en/quick-start-guides/10.7/arcgis-engine-developer-kit-and-engine-quick-start-guide.htm).

To achieve this goal, field data and soil surveys were collected from the dry grasslands of Banni. The investigation was carried out during the dry season at a total of forty-five grassland locations. The coordinates and altitudes of the research areas were recorded on the spot using a handheld Garmin 72H global positioning system. The bioclimate of the survey sites varies from hyperarid to arid. The soil sampling depth was fixed to 50 cm. ArcGIS 10.7 was utilised to a generate 10 × 10 km (Fig. 2) remote sensing grid and transects at each research location with a subgrid of approximately 5 × 5 km. The research area’s elevations were considered while choosing the sampling locations to minimise the potential effects of different microclimates driven by tectonics, aspect, and slope. Soil samples were sun-dried from the designated sampling locations before testing. Using a 2 mm filter, the samples were homogenised to remove any large soil particles or roots. Next, the physicochemical content of the soils was examined. The standardised standard protocols, as described by Alef and Nannipieri, were followed for the analysis of the physicochemical characteristics of the soils^51^. Using a digital electrical conductivity (EC) meter and a glass electrode, the samples were examined for electrical conductivity and pH in a 1:2 (w/v) soil solution^52^ and the water holding capacity was obtained using the gravimetric method^53^. Keen’s cup method^53^ was applied for the determination of soil bulk density. The organic carbon (SOC) in the soil was determined using the titrimetric determination method of Walkley and Black, 1934^54^. The soil texture was determined using the soil sieve method according to the USDA soil texture classification. All analyses were carried out in triplicate. Moreover, soils were examined for specific micronutrients, including iron (Fe), zinc (Zn), manganese (Mn), and copper (Cu), in accordance with earlier research describing the presence of various micronutrients and their mechanisms of action in diverse grasslands. An atomic absorption spectrophotometer (AAS) was used for micronutrient analysis. Soil extracts were combined with DTPA (diethylene tri amine pentaacetic acid) at a 1:2 ratio for the digestion of trace metals. AAS was used to evaluate the extracts at a flame temperature of 2950 °C. The equation below was used to calculate all of the minor nutrients.

1 $$ {\text{N}}_{{\text{c}}} = {\text{(Sr}} - {\text{Br) }} \times {\text{ soil}} - {\text{DTPA ratio }} \times {\text{ dilution factor,}} $$

Where Nc is the nutrient concentration in ppm, Sr is the sample reading and Br is the blank reading of the sample.

For available phosphorus, Olsen’s method for neutral alkaline soil^55^ was adopted. The total P in the soil samples was extracted by a mixture of concentrated sulfuric acid, hydrofluoric acid and hydrogen peroxide^56^, and the P concentration of the extract was determined using the same method as for available P. Potassium and sodium concentrations were determined by the flame photometer method^57^. We used the alkaline KMnO_4_ method with auto Kjeldahl to determine the available nitrogen in the study area^58^. A double-ring infiltrometer was used to measure the rate of soil infiltration (the measurements were made in accordance with the ASTM D3385-03 standard test methodology)^59^.We have taken care to have at least one test for each soil type and have optimised the number of locations for conducting infiltration tests based on soil type. When choosing the locations, geomorphology, topography, and land use are considered. The research area was divided into 15 infiltration locations, each of which contained a distinct soil type. The double-ring infiltrometer has inner and outer rings that are 30 and 60 cm in diameter, respectively. They were placed on the surface of the earth and securely attached 10 cm below the surface. As long as there was no more water infiltration, the tests were conducted. Eq. (2) was used to estimate infiltration rates:

2 $$ Infiltration\,\,\, Rate \left( {Ir} \right) = \frac{{\text{a}}}{{\text{b}}} \times 60{\text{ mm/hr}} $$

3 $$ {\text{Area of the ring (A) = 3}}{.142 } \times { (15)}^{2} {\text{ cm}}^{2} $$

Where *a* represents the rate of infiltration in millimeters, which is calculated as the volume of water delivered to the inner ring, and *b* represents the interval of time (in minutes) between two successive readings.

The Ca^++^ and Mg^++^ contents in the soils were estimated through the standard versenate method (EDTA complexometric titration) from water and ammonium acetate extracts at a 1:5 ratio. Na and K were analysed through flame photometry from water and ammonium acetate extracts at 1:5 ratios^60^. The values of the water extracts were subtracted from the values of the ammonium acetate extracts to calculate the corresponding net exchangeable values. The results were subjected to calculations of the SAR (sodium adsorption ratio) and CEC (cation exchange capacity). The SAR and CEC were calculated through the following equations:

4 $$ SAR = {\raise0.7ex\hbox{${Na^{ + } }$} \!\mathord{\left/ {\vphantom {{Na^{ + } } {\sqrt {\frac{{{\text{Ca}}^{ + + } + Mg^{ + + } }}{2}} }}}\right.\kern-0pt} \!\lower0.7ex\hbox{${\sqrt {\frac{{{\text{Ca}}^{ + + } + Mg^{ + + } }}{2}} }$}} $$

5 $$ {\text{CEC }} = {\text{ Na}}^{ + } + {\text{ K}}^{ + } + {\text{ Ca}}^{ + + } + {\text{ Mg}}^{ + + } $$

Additionally, Sentinel-2 satellite data with a spatial resolution of 30 m were utilised to generate the land use and land cover map, geomorphology, and land surface temperature. Slope maps were produced using DEM data from ASTER at a resolution of 1 arc second. The rainfall data were sourced from the Indian Meteorological Department. We excluded geological data because study area a has uniform features known as the Banni plains. Table 1 shows the spatial characteristics of the data employed in the study. To compare all the parameters with further field measurements, all the parameters were recorded. The geographic distribution may be determined by comparing the parameters from several field measurements. These well-defined sites are crucial for comprehending the spatial relationships and insight mechanisms of the processes affecting the suitability and dynamics of grasslands. Additionally, interpolation of these factors will reveal details on the spatial variations in the Banni Plain. Maps of the collected data were generated using inverse distance weighted (IDW) techniques. The IDW interpolation clearly assumes that nearby objects are more similar than those farther away^61^. The IDW is given by:

6 $$ z_{j} = k_{j} \mathop \sum \limits_{i = 1}^{n} \frac{1}{{d_{ij} }}Z_{i} $$

7 $$ k_{j} = \mathop \sum \limits_{i = 1}^{n} \frac{1}{{d_{ij} }} $$

**Table 1:** Data utilised in the research and their sources.

Sl. No	Parameters	Data	Sources	Resolution
1	LULC, geomorphology	Satellite imagery (sentinel-2)	ESA (https://scihub.copernicus.eu/dhus/#/home)	30 m
2	Slope	ASTER DEM	USGS (www.earthexplorer.usgs.gov)	30 m
3	N, P, K, SOM, bulk density, WHC, infiltration rate, Fe, Mn, Zn, Cu, soil texture, pH, EC, SAR, CEC, ESP Ca^++^ and Mg^++^	Soil samples (10 × 10 km grid)	Field survey and laboratory analysis	
4	Precipitation	Net cdf	Indian meteorological department	0.25° × 0.25°

The adjustment k_j_ in this formula ensures that the weights total up to 1. Z_i_ is the value of an established point. The distance between known points is d_ij_. The unknown point is Z_j,_ andn is a user-selectable exponent.

Using the Arc GIS 10.7, Erdas 9.2 and Idrisi Selva image processing techniques, the satellite imagery bands were combined, fused, and layered. A traditional coordination system was applied to all of these factors, and projection and weighted overlay analysis (WOA) were performed.

### Multi criteria decision analysis using GIS techniques

**Figure 3:**
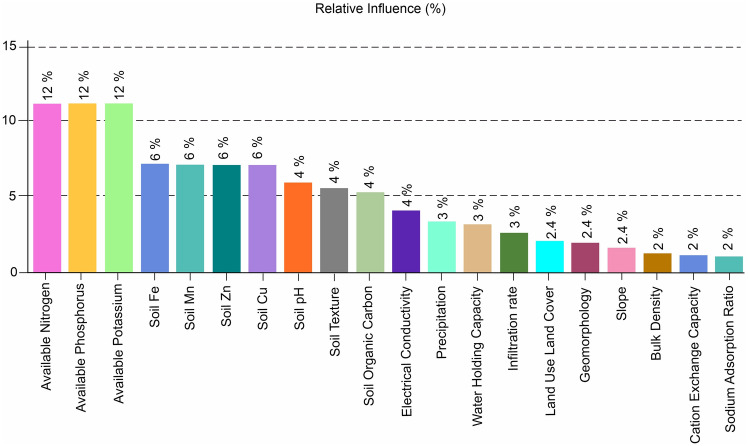
Parameters weights of relative importance.

For identifying grassland suitability zones, multi criteria decision analysis using the analytical hierarchical process (AHP) is the most widely used and well-known GIS-based method. The integration of all theme levels is facilitated by this method. Twenty distinct theme levels were considered for this investigation. The dynamics of the area's grasslands are thought to be governed by these 20 thematic levels. These influencing factors are weighted according to their interactions with expert evaluations, terrain, climate, and edaphic characteristics. A layer that has a significant influence on the suitability of grasslands is indicated by a high weight value, whereas a layer that has little impact is indicated by a low weight parameter. The weights allocated to each parameter (Fig. 3) were determined by applying Saaty’s relative importance value scale (1–9). Furthermore, the weights were assigned by taking into account the review of past research and field experience.

The Saaty's scale of relative importance suggests that a value of 9 indicates extreme significance; 8, very strong; 7, very to extreme importance; 6, strong plus; 5, strong importance; 4, moderate plus; 3, moderate importance; 2, weak importance; and 1, equal importance. Thematic layers are classified according to their relevance, and weights are allocated to them accordingly. To compare all of the theme layers with one another, a pairwise comparison matrix (Table 2) was used. To assign weights, thematic layer subclasses were reclassified using the natural breaks classification method of the GIS platform. The subclasses of each theme layer were given a score between 0 and 9 according to how each affected the suitability and productivity of the grasslands^28^. The ranked and weighted thematic layers are shown in Table 3. The following procedures were used to calculate the consistency ratio (CR): (1) the principal eigenvalue (ʎ) was computed by the eigenvector technique (Table 4), and (2) the consistency index (CI) was calculated from the equation given below:

**Table 2:** Pairwise comparison matrix table of twenty thematic layers chosen for the present study.

Factors	A.W	N	P	K	Fe	Mn	Zn	Cu	pH	ST	SOC	EC	PP	WHC	In	LULC	Geo	SL	BD	CEC	SAR	GM	NW
N	9	9/9	9/9	9/9	9/8	9/8	9/8	9/8	9/7	9/7	9/7	9/7	9/6	9/6	9/6	9/5	9/5	9/5	9/4	9/4	9/4	2.44	0.1222
P	9	9/9	9/9	9/9	9/8	9/8	9/8	9/8	9/7	9/7	9/7	9/7	9/6	9/6	9/6	9/5	9/5	9/5	9/4	9/4	9/4	2.44	0.1222
K	9	9/9	9/9	9/9	9/8	9/8	9/8	9/8	9/7	9/7	9/7	9/7	9/6	9/6	9/6	9/5	9/5	9/5	9/4	9/4	9/4	2.44	0.1222
Fe	8	8/9	8/9	8/9	8/8	8/8	8/8	8/8	8/7	8/7	8/7	8/7	8/6	8/6	8/6	8/5	8/5	8/5	8/4	8/4	8/4	1.22	0.0611
Mn	8	8/9	8/9	8/9	8/8	8/8	8/8	8/8	8/7	8/7	8/7	8/7	8/6	8/6	8/6	8/5	8/5	8/5	8/4	8/4	8/4	1.22	0.0611
Zn	8	8/9	8/9	8/9	8/8	8/8	8/8	8/8	8/7	8/7	8/7	8/7	8/6	8/6	8/6	8/5	8/5	8/5	8/4	8/4	8/4	1.22	0.0611
Cu	8	8/9	8/9	8/9	8/8	8/8	8/8	8/8	8/7	8/7	8/7	8/7	8/6	8/6	8/6	8/5	8/5	8/5	8/4	8/4	8/4	1.22	0.0611
pH	7	7/9	7/9	7/9	7/8	7/8	7/8	7/8	7/7	7/7	7/7	7/7	7/6	7/6	7/6	7/5	7/5	7/5	7/4	7/4	7/4	0.81	0.0407
ST	7	7/9	7/9	7/9	7/8	7/8	7/8	7/8	7/7	7/7	7/7	7/7	7/6	7/6	7/6	7/5	7/5	7/5	7/4	7/4	7/4	0.81	0.0407
SOC	7	7/9	7/9	7/9	7/8	7/8	7/8	7/8	7/7	7/7	7/7	7/7	7/6	7/6	7/6	7/5	7/5	7/5	7/4	7/4	7/4	0.81	0.0407
EC	7	7/9	7/9	7/9	7/8	7/8	7/8	7/8	7/7	7/7	7/7	7/7	7/6	7/6	7/6	7/5	7/5	7/5	7/4	7/4	7/4	0.81	0.0407
PP	6	6/9	6/9	6/9	6/8	6/8	6/8	6/8	6/7	6/7	6/7	6/7	6/6	6/6	6/6	6/5	6/5	6/5	6/4	6/4	6/4	0.61	0.0306
WHC	6	6/9	6/9	6/9	6/8	6/8	6/8	6/8	6/7	6/7	6/7	6/7	6/6	6/6	6/6	6/5	6/5	6/5	6/4	6/4	6/4	0.61	0.0306
In	6	6/9	6/9	6/9	6/8	6/8	6/8	6/8	6/7	6/7	6/7	6/7	6/6	6/6	6/6	6/5	6/5	6/5	6/4	6/4	6/4	0.61	0.0306
LULC	5	5/9	5/9	5/9	5/8	5/8	5/8	5/8	5/7	5/7	5/7	5/7	5/6	5/6	5/6	5/5	5/5	5/5	5/4	5/4	5/4	0.49	0.0244
GM	5	5/9	5/9	5/9	5/8	5/8	5/8	5/8	5/7	5/7	5/7	5/7	5/6	5/6	5/6	5/5	5/5	5/5	5/4	5/4	5/4	0.49	0.0244
SL	5	5/9	5/9	5/9	5/8	5/8	5/8	5/8	5/7	5/7	5/7	5/7	5/6	5/6	5/6	5/5	5/5	5/5	5/4	5/4	5/4	0.49	0.0244
BD	4	4/9	4/9	4/9	4/8	4/8	4/8	4/8	4/7	4/7	4/7	4/7	4/6	4/6	4/6	4/5	4/5	4/5	4/4	4/4	4/4	0.41	0.0204
CEC	4	4/9	4/9	4/9	4/8	4/8	4/8	4/8	4/7	4/7	4/7	4/7	4/6	4/6	4/6	4/5	4/5	4/5	4/4	4/4	4/4	0.41	0.0204
SAR	4	4/9	4/9	4/9	4/8	4/8	4/8	4/8	4/7	4/7	4/7	4/7	4/6	4/6	4/6	4/5	4/5	4/5	4/4	4/4	4/4	0.41	0.0204

**Table 3:** Categorisation of factors influencing grassland suitability and the ranks and weights of thematic layers.

SL. No	Factors	Weight	Rank	Over all	Area. sq. km
1	Available Nitrogen				
	High	12	5	60	25.66
	Medium		4	48	60.46
	Low		3	36	169.97
	Very low		2	24	2356.26
2	Available Phosphorus				
	High	12	5	60	115.17
	Medium		4	48	704.04
	Low		3	36	1052.59
	Very low		2	24	740.33
3	Available Potassium				
	High	12	5	60	34.10
	Medium		4	48	162.98
	Low		3	36	1235.55
	Very low		2	24	1179.45
4	Soil Fe				
	High	06	5	30	64.65
	Medium		4	24	603.06
	Low		3	18	766.36
	Very low		2	12	1178.26
5	Soil Mn				
	High	06	5	30	113.81
	Medium		4	24	558.03
	Low		3	18	853.91
	Very low		2	12	1086.52
6	Soil Zn				
	High	06	5	30	40.86
	Medium		4	24	488.87
	Low		3	18	1350.81
	Very low		2	12	731.36
7	Soil Cu				
	High	06	5	30	235.76
	Medium		4	24	1244.30
	Low		3	18	768.60
	Very low		2	12	363.59
8	Soil pH				
	Very high	04	2	08	549.19
	High		3	12	908.20
	Medium		4	16	617.23
	Low		5	20	537.69
9	Soil texture				
	Loamy	04	5	20	234.82
	Silt loam		4	16	1170.72
	Clay loam		5	20	599.10
	Silt clay		4	16	370.21
	Sandy loam		3	12	141.23
	Loamy sand		3	12	75
	Silt		2	08	20.66
10	Soil organic carbon				
	High	04	5	20	536.08
	Medium		4	16	771.79
	Low		3	12	851.62
	Very low		2	08	453.03
11	EC				
	Very High	04	2	08	2031.89
	High		3	12	512.83
	Medium		4	16	48.40
	Low		5	20	18.91
12	Annual precipitation				
	High	03	5	15	1151.25
	Medium		4	12	923.57
	Low		3	09	539.02
13	Water holding capacity				
	Very High	03	5	15	317.44
	High		4	12	1267.68
	Medium		3	09	783.56
	Low		2	06	243.19
14	Infiltration rate				
	High	03	5	15	525.83
	Medium		4	12	1583.64
	Low		3	09	502.84
15	Land use land cover				
	Dense grassland	024	5	10	356.46
	Sparse grassland		4	08	887.31
	Water bodies		5	10	8.58
	Barren land		3	06	579.94
	Agriculture		5	10	471.69
	Salt intruded areas		2	04	148.45
	Built up areas		1	02	58.24
	Others		2	04	102.03
16	Geomorphology				
	High level mudflats	024	5	10	344.23
	Low level mudflats with saline depressions		3	06	583.47
	Mid level mudflats		5	10	1685.14
	Lineaments		4	08	–
	Shallow gullies		5	10	–
17	Slope				
	Moderate	024	3	06	6.94
	Gentle		4	08	73.38
	Flat		5	10	2518.41
18	Soil bulk density				
	High	02	3	06	478.88
	Medium		4	08	944.67
	Low		5	10	1188.48
19	Cation exchange capacity				
	High	02	5	10	53.76
	Medium		4	08	743.74
	Low		3	06	1815.07
20	Sodium adsorption ratio				
	High	02	3	06	348.02
	Medium		4	08	840.51
	Low		5	10	1423.43

**Table 4:** Results of the consistency ratio.

Factors	N	P	K	Fe	Mn	Zn	Cu	pH	ST	SOC	EC	PP	WHC	In	LULC	Geo	SL	BD	CEC	SAR	WS	λ
N	0.122	0.122	0.122	0.122	0.122	0.122	0.122	0.122	0.122	0.122	0.122	0.122	0.122	0.122	0.122	0.122	0.122	0.122	0.122	0.122	2.444	20
P	0.122	0.122	0.122	0.122	0.122	0.122	0.122	0.122	0.122	0.122	0.122	0.122	0.122	0.122	0.122	0.122	0.122	0.122	0.122	0.122	2.444	20
K	0.122	0.122	0.122	0.122	0.122	0.122	0.122	0.122	0.122	0.122	0.122	0.122	0.122	0.122	0.122	0.122	0.122	0.122	0.122	0.122	2.444	20
Fe	0.061	0.061	0.061	0.061	0.061	0.061	0.061	0.061	0.061	0.061	0.061	0.061	0.061	0.061	0.061	0.061	0.061	0.061	0.061	0.061	1.222	20
Mn	0.061	0.061	0.061	0.061	0.061	0.061	0.061	0.061	0.061	0.061	0.061	0.061	0.061	0.061	0.061	0.061	0.061	0.061	0.061	0.061	1.222	20
Zn	0.061	0.061	0.061	0.061	0.061	0.061	0.061	0.061	0.061	0.061	0.061	0.061	0.061	0.061	0.061	0.061	0.061	0.061	0.061	0.061	1.222	20
Cu	0.061	0.061	0.061	0.061	0.061	0.061	0.061	0.061	0.061	0.061	0.061	0.061	0.061	0.061	0.061	0.061	0.061	0.061	0.061	0.061	1.222	20
pH	0.041	0.041	0.041	0.041	0.041	0.041	0.041	0.041	0.041	0.041	0.041	0.041	0.041	0.041	0.041	0.041	0.041	0.041	0.041	0.041	0.815	20
ST	0.041	0.041	0.041	0.041	0.041	0.041	0.041	0.041	0.041	0.041	0.041	0.041	0.041	0.041	0.041	0.041	0.041	0.041	0.041	0.041	0.815	20
SOC	0.041	0.041	0.041	0.041	0.041	0.041	0.041	0.041	0.041	0.041	0.041	0.041	0.041	0.041	0.041	0.041	0.041	0.041	0.041	0.041	0.815	20
EC	0.041	0.041	0.041	0.041	0.041	0.041	0.041	0.041	0.041	0.041	0.041	0.041	0.041	0.041	0.041	0.041	0.041	0.041	0.041	0.041	0.815	20
PP	0.031	0.031	0.031	0.031	0.031	0.031	0.031	0.031	0.031	0.031	0.031	0.031	0.031	0.031	0.031	0.031	0.031	0.031	0.031	0.031	0.611	20
WHC	0.031	0.031	0.031	0.031	0.031	0.031	0.031	0.031	0.031	0.031	0.031	0.031	0.031	0.031	0.031	0.031	0.031	0.031	0.031	0.031	0.611	20
In	0.031	0.031	0.031	0.031	0.031	0.031	0.031	0.031	0.031	0.031	0.031	0.031	0.031	0.031	0.031	0.031	0.031	0.031	0.031	0.031	0.611	20
LULC	0.024	0.024	0.024	0.024	0.024	0.024	0.024	0.024	0.024	0.024	0.024	0.024	0.024	0.024	0.024	0.024	0.024	0.024	0.024	0.024	0.489	20
GM	0.024	0.024	0.024	0.024	0.024	0.024	0.024	0.024	0.024	0.024	0.024	0.024	0.024	0.024	0.024	0.024	0.024	0.024	0.024	0.024	0.489	20
SL	0.024	0.024	0.024	0.024	0.024	0.024	0.024	0.024	0.024	0.024	0.024	0.024	0.024	0.024	0.024	0.024	0.024	0.024	0.024	0.024	0.489	20
BD	0.020	0.020	0.020	0.020	0.020	0.020	0.020	0.020	0.020	0.020	0.020	0.020	0.020	0.020	0.020	0.020	0.020	0.020	0.020	0.020	0.407	20
CEC	0.020	0.020	0.020	0.020	0.020	0.020	0.020	0.020	0.020	0.020	0.020	0.020	0.020	0.020	0.020	0.020	0.020	0.020	0.020	0.020	0.407	20
SAR	0.020	0.020	0.020	0.020	0.020	0.020	0.020	0.020	0.020	0.020	0.020	0.020	0.020	0.020	0.020	0.020	0.020	0.020	0.020	0.020	0.407	20

ʎ_max_ = 400/20 = 208Where n is the number of factors used in the analysis.

CI = (20−20)/(20−1) = 0.

The consistency ratio is defined as CR = CI/RCI, where RCI = random consistency index value, whose values were obtained from Saaty’s standard^62^ (Table 5).

**Table 5:** Saaty’s ratio index for different values of N.

The consistency indices of randomly generated reciprocal matrices^62^										
Order of the matrix										
N	1	2	3	4	5	6	7	8	9	10
RCI value	0.00	0.00	0.58	0.90	1.12	1.24	1.32	1.41	1.45	1.49
N	11	12	13	14	15	16	17	18	19	20
RCI Value	1.51	1.53	1.56	1.57	1.59	1.6	1.61	1.61	1.62	1.63

CR = 0/1.63 = 0.

According to Saaty^62^, a CR of 0.10 or less is sufficient to continue the analysis. If the consistency value is greater than 0.10, the judgement must be reviewed to identify the root reasons for the inconsistency and to make the necessary corrections. If the CR is 0, then the pairwise comparison has complete consistency. The judgement matrix is relatively consistent because the threshold value is not higher than 0.1. The consistency ratios of all the thematic layers are shown in Table 4.

To generate a map of the grassland suitability of the Banni Plain, all twenty thematic layers were integrated with the weighted overlay analysis method in the GIS platform using Eq. (9):

9 $$ GSZ = \mathop \sum \limits_{{\text{i}}}^{{\text{n}}} \left( {{\text{X}}_{{\text{A}}} \times Y_{B} } \right) $$

Where GSZ = Grassland suitability zones, X- represents the weight of the thematic layers, and Y- represents the rank of the thematic layer sub-class. A (A = 1, 2, 3, ……, X) represents the thematic map, and B (B = 1, 2, 3, ……, Y) represents the thematic map class.

**Figure 4:**
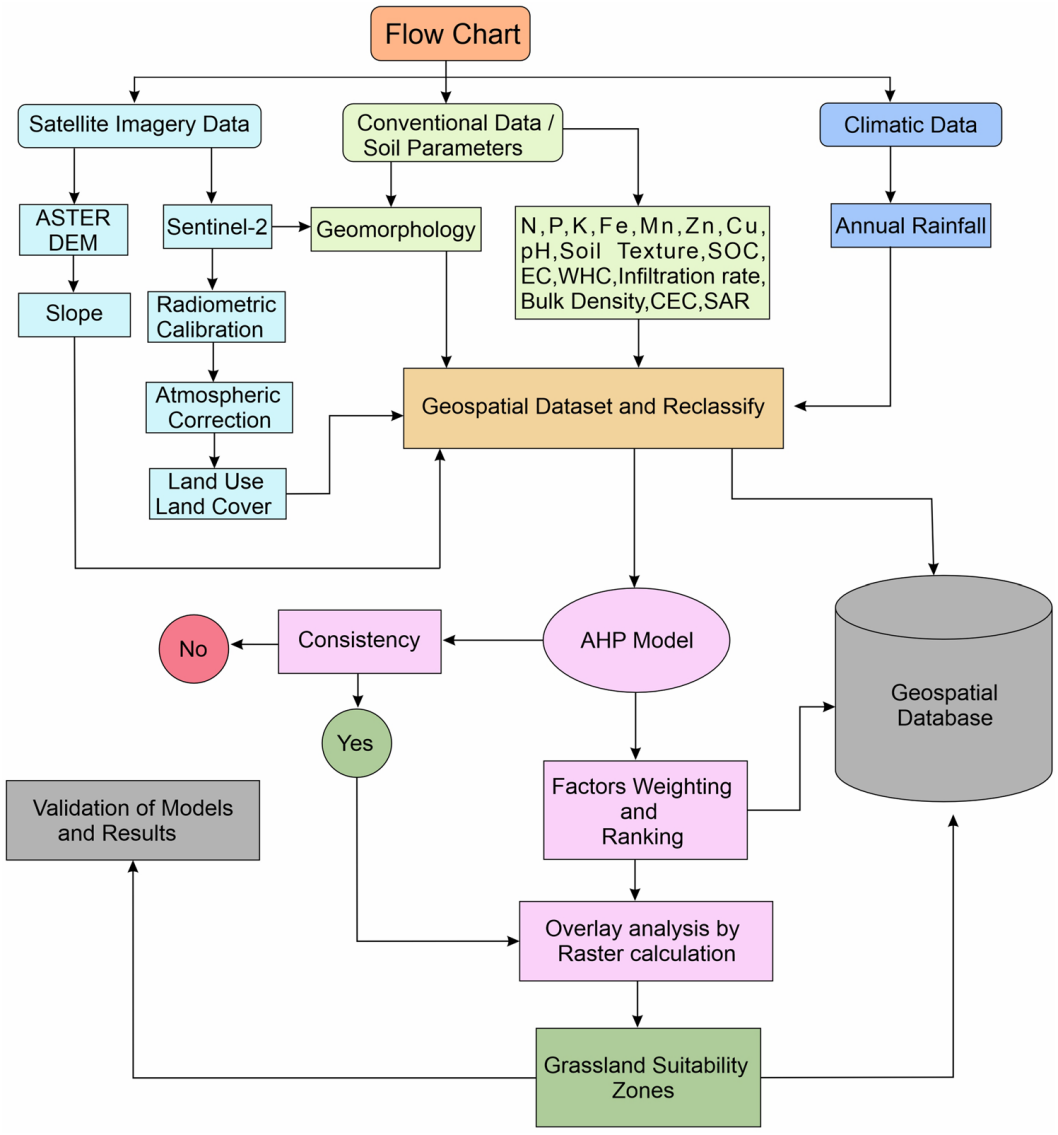
Methodology flow chart.

The final grassland suitability zone map was classified into five zones: not suitable, marginally suitable, moderately suitable, suitable, and highly suitable^28^. The final result was evaluated using data from scientific experts and a field survey conducted for the current investigation. A flow chart of the approach used in this investigation is shown in Fig. 4.

### Statistical analysis

The sets of data derived from the aforementioned investigations were subjected to a number of statistical tests to determine whether the variations in soil physicochemical characteristics and climatic, topographic, and nutrient concentrations across the Banni Grassland were significant. First, the Shapiro‒Wilk normality (p ≤ 0.05) test was performed on the datasets to ensure that they were not normally distributed. Additionally, the Kruskal–Wallis *H* test was selected as the non-parametric test that could be used on the data to identify significant differences across the different divisions. Statistical analysis was conducted using the following statistical software: IBM SPSS version 26, SAS version 9.3, Microsoft Excel version 7, and Origin Pro 2022.

## Results and discussion

### Soil nitrogen

**Figure 5:**
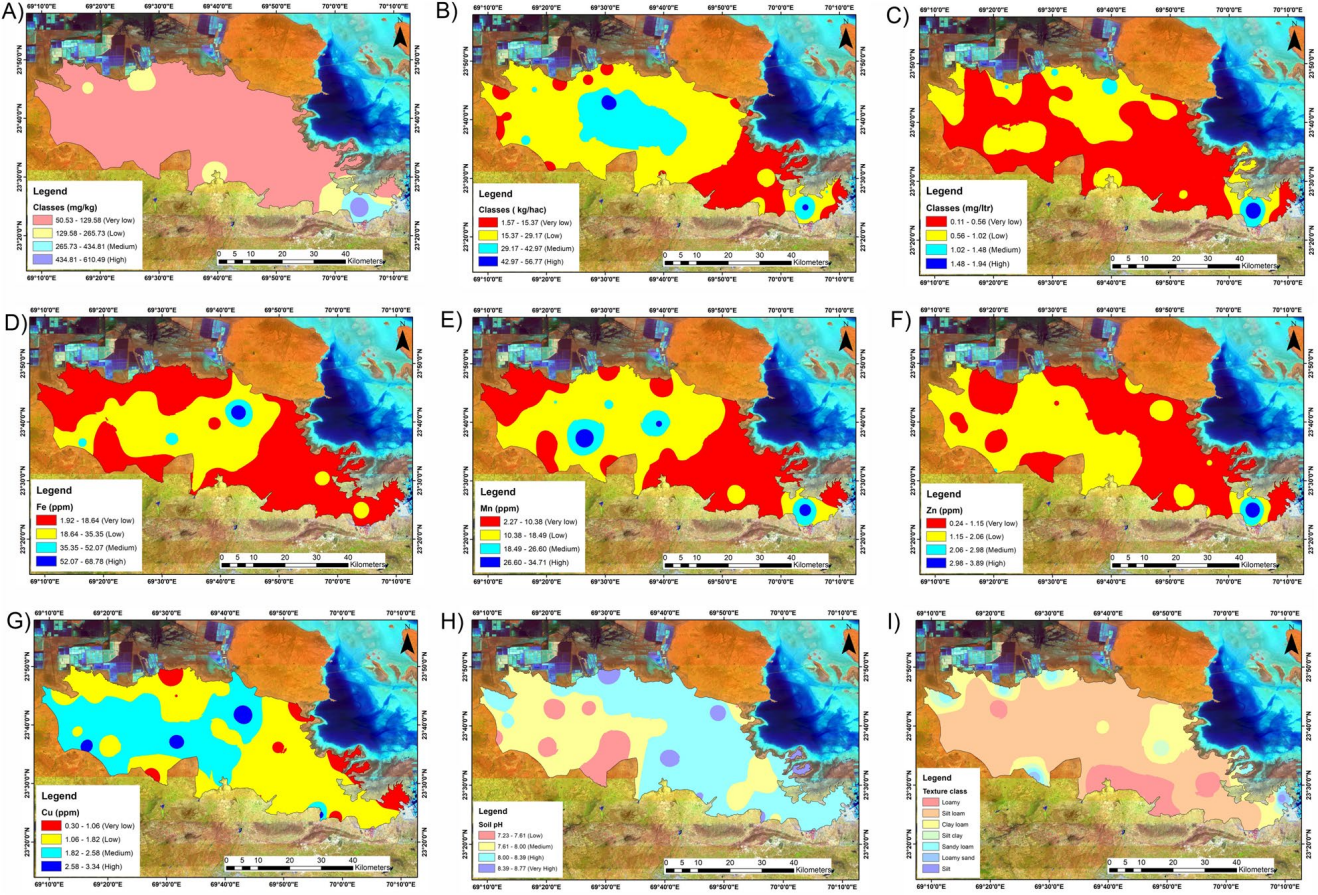
Spatial map of parameters. (**A**): Soil N (mg/kg); (**B**): Soil P (kg/hac); (**C**): Soil K (mg/ltr); (**D**): Fe (ppm); (**E**): Mn (ppm); (**F**): Zn (ppm); (**G**): Cu (ppm); (**H**): Soil pH; (**I**) Soil Texture. The map prepared by authors using Arc GIS version 10.7 (https://desktop.arcgis.com/en/quick-start-guides/10.7/arcgis-engine-developer-kit-and-engine-quick-start-guide.htm).

Nitrogen is a crucial nutrient for plants since it is one of the major nutrients required in high proportions for plant metabolism and development and is the most limiting factor for grassland productivity^63,64^. Atmospheric nitrogen is the principal source of nitrogen in the soil. Before it can be used in the soil, it must be converted from its natural state as N_2_ in the atmosphere. Although nitrogen is naturally present in the soil in organic forms as residues of plants and animals, it is not readily available to plants and must first undergo transformation by microbes for them to use it^65^. The inorganic forms NH_4_^+^ and NO_3_^−^(sometimes referred to as mineral nitrogen) make up the majority of the nitrogen that is accessible to plants. Nitrogen is often sluggishly released from soil minerals and only provides a small amount of nitrogen to the soil. The range of soil nitrogen (N) in the research region is 50.53 to 610.49 mg/kg (Fig. 5A), with the bulk falling in the very low category (50.53 to 129.58 mg/kg) and spanning an area of approximately 2356.20 sq. km, or approximately 90.19% of the land area. (Fig. 4b). Compared to 169.97 sq. km of low N (2.31%) and 60.46 sq. km (6.50%) of medium N concentrations, only 25.66 sq. km (0.98%) of the region is covered by high available N concentrations.

### Soil phosphorus

Second only to nitrogen, phosphorus (P) is a major limiting nutrient in agriculture and is removed from arable and grassland soils by crop and grass withdrawals and erosion^66^. The main function of phosphorus in plants is to store and transfer energy produced during photosynthesis for use in growth and imination. Numerous processes include P cycling in the soil. Adequate P promotes root development, winter hardiness, tillering, and maturity. The soil phosphate concentration is a good indicator of P cycling in soils and a crop’s response to P fertiliser. The rate of P mineralisation from the decomposition of organic matter is influenced by site and climate factors such as salinity, moisture, and soil aeration. In well-aerated soils (where oxygen concentrations are greater), phosphorus is released more quickly, whereas in saturated, wet soils, it is released much more slowly^63^. The soil P in the study region ranged from 1.57 to 56.77 kg/hac (Fig. 5B), with the majority falling between 15.37 and 29.17 kg/hac (low category) and spanning an area of approximately 1052.59 sq. km, or approximately 40.29% of the land area. (Fig. 4b). In contrast to the 740.33 sq. km with very low P (28.34%) and 704.04 sq. km with a moderate P concentration (26.95%), only 115.17 sq. km (4.40%) of the region is coated by high P concentrations.

### Soil potassium

Together with nitrogen and phosphorus, potassium (K) is one of the three major plant macronutrients that are consumed relatively significantly by plants from soil. Potassium increases grassland productivity and improves product quality^63^. Additionally, potassium aids plants in overcoming environmental challenges such as disease, insect attacks, and cold and drought stress. It facilitates the development of a strong and healthy root system and increases the effectiveness of nutrient uptake and usage, particularly for nitrogen. Furthermore, cattle nutrition depends on potassium. Potassium (K) is an essential nutrient for plant growth. N, P, and K all have impacts on grassland production, with N and P acting as the major limiting factors and K acting as the secondary limiting factor. Among the functional groups, P is the most limiting factor, followed by N and P for grasses, P for forbs, and all three for shrubs^67^. The range of soil K concentrations in the study area is 0.11–1.94 mg/L (Fig. 5C). Generally, the K content was lower in all the samples. However, the K was moderate to fairly acceptable in some patches of the research site. The K content in the soil, however, suggests that the available K in the soil is low when compared to the approved limits. Approximately 45.15% (1179.45 sq. km) of the land had available K contents in the range of 0.11 to 0.56 (mg/ltr), 47.30% (1235.55 sq. km) had available K contents in the range of 0.56 to 1.02 (mg/ltr), 6.23% (162.98 sq. km) had available K contents in the range of 1.02 to 1.48 (mg/ltr), and 1.30% (34.10 sq. km) had available K contents in the range of 1.48 to 1.94 (mg/ltr).

### Iron (Fe)

Minute amounts of micronutrients, sometimes referred to as trace minerals, are needed by plants and animals. The main heavy metals include copper (Cu), zinc (Zn), manganese (Mn), and iron (Fe). This, however, does not imply that their contribution is insignificant. Their absence, for instance, might result in severe crop productivity issues in forages and health issues in cattle^68^. Fe is necessary for the production of chlorophyll and photosynthesis in plants. The distribution of plant species in natural ecosystems is determined by soil iron availability, which also restricts crop output and nutrient quality. The Fe concentrations ranged from 1.92 to 68.78 ppm (Fig. 5D) in the study region, with the majority ranging between 1.92 and 18.64 and covering 1178.26 sq. km (or 45.1%) of the land. A total of 766.36 sq. km (29.3%) are covered by an area with low Fe in the range of approximately 18.64 to 35.35 ppm. Similarly, high Fe, which ranges from 52.07 to 68.78 ppm, and medium Fe (35.35 to 52.07 ppm) cover an area of approximately 64.65 sq. km (2.4%) and 603.06 sq. km (23.08%), respectively.

### Manganese (Mn)

In addition to assisting metabolic processes in several plant cell compartments, manganese (Mn) is an essential element for plant growth and development^69^. It also plays a significant role in the activation of various enzymes involved in the citric acid cycle, oxidation processes, carboxylation, carbohydrate metabolism, and phosphorus reactions^70^. In sandy soils with a high pH and a high level of organic matter, manganese reactions are most likely to occur^68^. Mn levels ranged from 2.27 to 34.71 ppm in the study region (Fig. 5E), with the majority ranging between 2.27 and 10.38 and covering 1086.52 sq. km (or 41.5%) of the land. A region with low Mn in the range of approximately 10.38 to 18.49 ppm covers 853.91 sq. km (32.6%) of the total area. Similarly, high Mn covers an area of approximately 113.81 sq. km (4.3%), and medium Mn covers an area of approximately 558.03 sq. km (21.3%), respectively. High Mn contents range from 26.60 to 34.71 ppm.

### Zinc (Zn)

Zinc is a crucial micronutrient for humans, animals, and plants. Zn plays a significant role in numerous enzymes that catalyse metabolic processes in plants. Additionally, zinc is important for photosynthesis, cell membrane integrity, protein synthesis, pollen development, and disease resistance in plants and increases the levels of antioxidant enzymes and chlorophyll in plant tissues^71^. Zn levels in the study region ranged from 0.24 to 3.89 ppm (Fig. 5F), with the majority occurring between 0.24 and 1.15 ppm and between 1.15 and 2.06 ppm, covering 731.36 sq. km (or 28%) and 1350.81 sq. km (or 51.7%) of the land, respectively. Similarly, high Zn, which ranges from 2.98 to 3.89 ppm, and medium Zn (2.06 to 2.98 ppm) cover an area of approximately 40.86 sq. km (1.5%) and 488.87 sq. km (18.7%), respectively.

### Copper (Cu)

Copper plays a vital role in several plant functions. It is essential for photosynthesis, enzyme activity, and the formation of lignin, a component of plant cell walls. Copper also aids in the uptake and utilisation of iron, promoting overall plant growth and development^70^. However, excessive copper in soil can be toxic to plants, causing damage to roots and inhibiting their ability to absorb water and nutrients. The Cu concentrations in the research area varied from 0.30 to 3.34 ppm (Fig. 5G), although most were between 1.06 and 1.82 ppm and between 1.82 and 2.58 ppm, respectively, encompassing 768.60 sq. km (or 29.4%) and 1244.30 sq. km (or 47.6%) of the land. Similarly, high Cu, which ranges from 2.58 to 3.34 ppm, and very low Cu (0.30 to 1.06 ppm) cover an area of approximately 235.76 sq. km (9.02%) and 363.59 sq. km (13.9%), respectively.

### Soil pH

One of the key elements affecting the overall composition of grassland ecosystems is the pH of the soil^72^. Soil pH has an impact on nutrient availability, plant growth, and crop output. The minerals that make up soil have a major role in determining how it responds. The availability of nutrients, phytotoxicity, and crop compatibility may all be affected by pH. The optimal pH range for the majority of plants is between 5.5 and 7.0. The alkaline pH is greater than 7, whereas the acidic pH is less than 7^73^. The parent material, organic matter content, and inorganic matter content are the key factors affecting grassland soil pH. For instance, quartz and feldspar are the primary sources of silt and have direct and indirect impacts on H^+^ ion accumulation, lowering the pH of grassland soils. Additionally, organic matter releases H^+^ ions into the soil and can lower the pH because it includes numerous acid functional groups that are a source of H^+^ ions in the soil. As the soil pH increases, the abundance of grassland species also decreases^72^. The soil samples from the research area had a pH that varied from 7.23 to 8.7 (Fig. 5H), with an average pH of approximately 8.04.The bulk of the research region, which comprises approximately 908.20 sq. km, or approximately 34.76% of the total area, has soils that are suitable for grassland and have a pH range of 8 to 8.39.In patches that encompassed an area of approximately 537.69 square kilometres, pH values ranging from 7.23 to 7.61 were also observed, with 20.58% of the land being best suited for the development of grasslands.

### Soil texture

Since soil texture is a crucial factor in the laws governing grassland ecosystems^74^, determining soil texture functioning zones requires a precise methodology. Numerous physical and chemical properties are directly influenced by soil texture, and these properties in turn influence soil fertility and productivity. Soil texture affects drainage and water retention in agriculture, which affects crop selection and irrigation methods^75^. Soil texture impacts biodiversity and plant communities in ecological settings, influencing habitat suitability and supporting several ecosystem processes, including grassland stability^74^. The most common soil textures in the study region are silt loam and clay loam, which span an area of approximately 1170.72 sq. km (44.82%) and 599.10 sq. km (22.93%), respectively (Fig. 5I). Similarly, loam, silty clay, sandy loam, loamy sand and silt cover an area of approximately 234.82 sq. km (8.99%), 370.21 sq. km (14.17%), 141.23 sq. km (5.40%), 75 sq. km (2.87%) and 20.66 sq. km (0.79%), respectively. The loamy and clay loam soils were given the highest rank owing to their water holding capacity, fertility, and nutrient enrichment, while loamy sand and silt were given the lowest rank due to their relatively low water holding capacity, fertility, and nutrient content.

### Soil organic carbon

**Figure 6:**
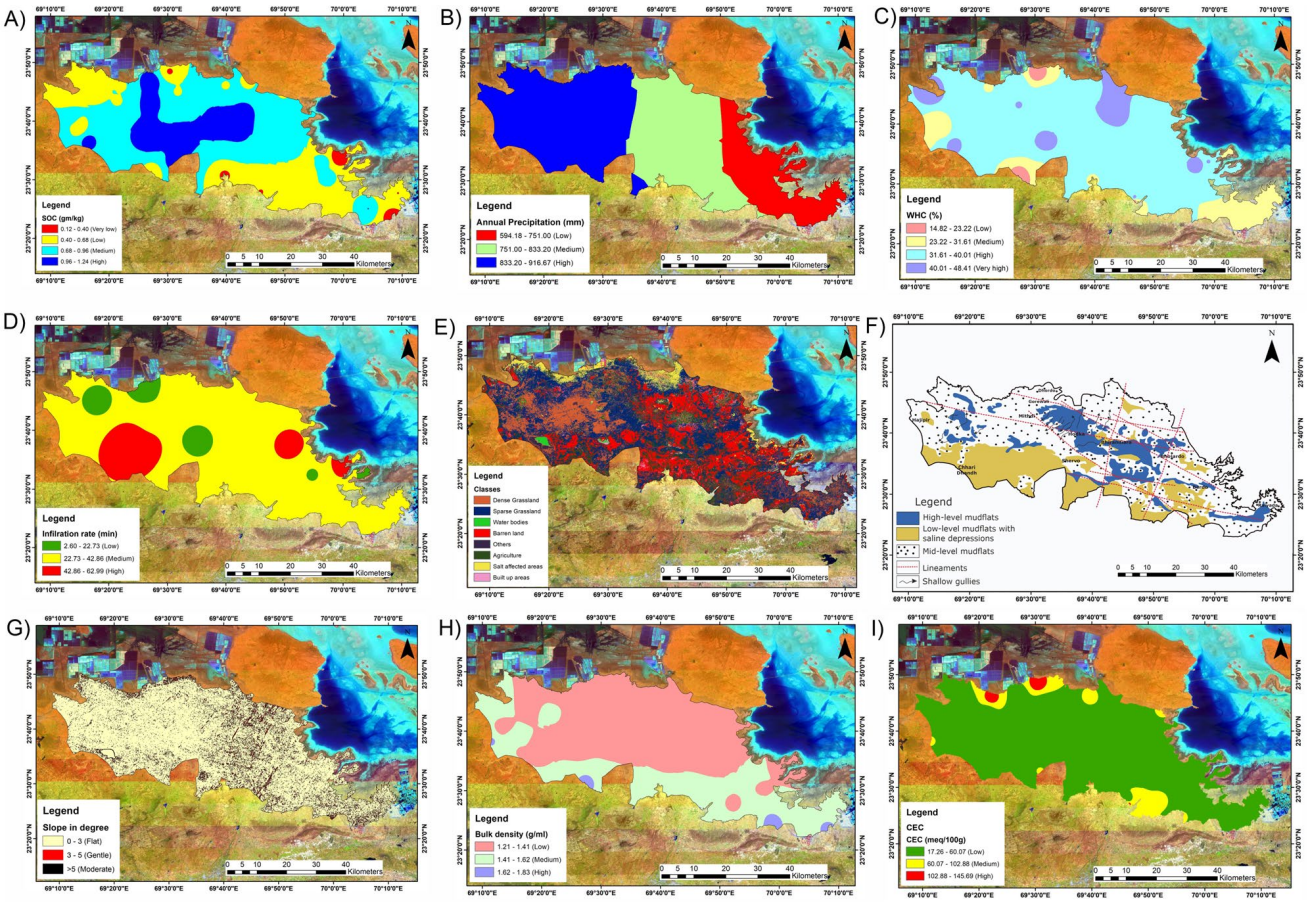
Spatial map of parameters. (**A**): Soil organic carbon (gm/kg); (**B**): Precipitation (mm); (**C**): WHC (%); (**D**): Infiltration rate (min); (**E**): LULC; (**F**): Geomorphology; 6G: Slope (°); (**H**): Bulk density (g/ml); (**I**): CEC (meq/100 g). The map prepared by authors using Arc GIS version 10.7 (https://desktop.arcgis.com/en/quick-start-guides/10.7/arcgis-engine-developer-kit-and-engine-quick-start-guide.htm).

The amount of carbon stored in soil organic matter is known as soil organic carbon (SOC). It plays a crucial role in controlling temperature, biomass production, water holding capacity, nutrient cycling, and soil fertility on Earth. Previous research has revealed that grassland soil carbon stores have been largely reduced worldwide as a result of improper usage or management but that management changes may also boost soil carbon stocks and prevent the deterioration of grasslands^76^. A thorough analysis revealed that the SOC content in the region's soils ranged from 0.12 to 1.24 gm/kg (Fig. 6A), with the bulk decreasing between 0.40 and 0.68 gm/kg and encompassing 851.62 sq. km (or 32.59%) of the land. A total of 453.03 sq. km (17.34%) are covered by an area with very low SOC in the range of approximately 0.12 to 0.40 gm/kg. Similarly, high SOC, which ranges from 0.96 to 1.24 gm/kg, and medium SOC (0.68 to 0.96 gm/kg) cover an area of approximately 536.08 sq. km (20.51%) and 771.79 sq. km (29.54%), respectively.

### EC

**Figure 7:**
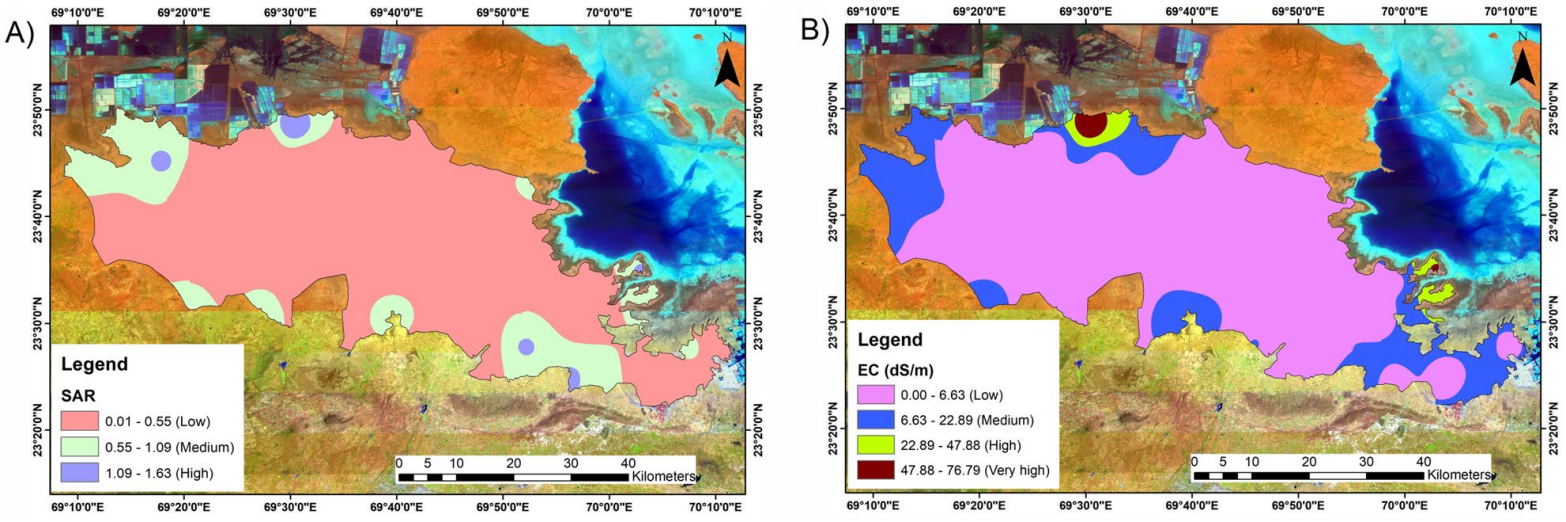
Spatial map of parameters. (**A**): SAR; (**B**): EC (dS/m). The map prepared by authors using Arc GIS version 10.7 (https://desktop.arcgis.com/en/quick-start-guides/10.7/arcgis-engine-developer-kit-and-engine-quick-start-guide.htm).

The electrical conductivity (EC) of a solution is a measure of its salt content and is an indicator of its electrolyte concentration. The number of ions available to plants in the root zone corresponds to the EC of the nutrient solution^74^. A higher EC typically lowers nutrient absorption by increasing nutrient solution osmotic pressure, wastes nutrients, and increases nutrient outflow into the environment, resulting in environmental pollution. A lower EC may have adverse effects on plant health and output. Most crops have an EC range of 2 to 3.5 mS/cm. Previous research has revealed that soil salinisation is linked to the degradation of semiarid and arid grasslands. The degradation of grasslands has also accelerated as a result of a negative feedback mechanism caused by changes in land cover and significant salinisation of the soil^77^. In the studied area, the EC varied from 0.11 to 76.79 dS/m (Fig. 7B), with the bulk decreasing between 0.11 and 6.63 dS/m and encompassing 2031.89 sq. km (or 77.7%) of the land. A total of 18.9 sq. km (0.72%) are covered by an area with very high salinity in the range of approximately 47.88 to 76.79 dS/m. Similarly, high salinity, which ranges from 22.89 to 47.88 dS/m, and medium salinity (6.63 to 22.89 dS/m) cover areas of approximately 48.40 (1.85%) sq. km and 512.83 (19.63%) sq. km, respectively.

### Precipitation

The primary water source in the hydrological cycle and the most important determining factor for a region’s groundwater is rainfall^40^. Precipitation timing and amount are the primary factors that most significantly influence the health of semiarid grassland ecosystems. In a semiarid grassland, the amount of green plants changes greatly from year to year in part because of precipitation^78^. The 2021 annual rainfall data from the IMD were utilised in this study. The yearly rainfall fluctuates between 1180 and 2157 mm (Fig. 6B). The rainfall was divided into three groups based on the maximum and minimum values: low (594.18–751 mm), medium (751–833.20 mm), and high (833.20–916.67 mm), covering areas of approximately 539.02 sq. km (20.62% of the area), 923.57 sq. km (35.3% of the area) and 1151.25 sq.km (44.04% of the area) respectively.

### Water holding capacity (WHC)

In semiarid grassland ecosystems, the amount of water in the soil is the main constraint on plant development and carbon cycling^79^. Monitoring water holding capacity is critical for successful water management in horticulture and agriculture. It helps determine the soil’s ability to hold water by providing crucial information for irrigation scheduling, water conservation, and other related tasks. By frequently monitoring water retention capacity, farmers and growers may reduce water waste and adverse environmental effects while improving irrigation practices, preventing waterlogging or drought stress, and increasing crop production^80^. The water holding capacity of the research region ranges from 14.82 to 48.41% (Fig. 6C). The low water holding capacity covers an area of approximately 243.19 sq. km (9.3% of the total area). Similarly, medium, high, and very high water holding capacities cover areas of approximately 783.56 (29.9% of the area), 1267.68 (48.5% of the area), and 317.44 sq. km (12.1% of the area), respectively.

### Infiltration rate

Soil water infiltration is the process by which irrigation or rainfall penetrates the soil either horizontally or vertically through pores, tying surface water, soil water, and groundwater together. Plants can only take up and use soil water through soil infiltration^81^. In dry and semiarid climates, this process is the primary impediment to the establishment of grasslands and ecological restoration. It is closely linked to soil erosion, runoff, and nutrient migration^82^. Rainfall duration and intensity both affect infiltration. High-intensity and short-duration rain causes less infiltration and more surface runoff, whereas low-intensity and long-duration rain results in more infiltration than does run-off^40^. The infiltration rate in the research region varied from 2.60 to 62.99 (Fig. 6D). A region of approximately 502.84 sq. km is covered by a low infiltration rate (2.60 to 22.73 min). Similarly, medium and high infiltration rates span areas of approximately 1583.64 sq. km (22.73 to 42.86 min) and 525.83 sq. km (42.86 to 62.99 min), respectively.

### Land use land cover (LULC)

Satellite imagery was used to better understand the specifics of land use and land cover classification (LULC), which is crucial for identifying potential land suitability restoration points of interest. LULC provides vital information on the infiltration rate, moisture in the soil, groundwater, surface water, and so on, as well as information on grassland requirements^40,83^. The land use/land cover map was classified into eight categories: built-up areas, water bodies, barren land, agricultural areas, salt-affected areas, dense grassland, sparse grassland, and barren land (Fig. 6E). As a result, 356.46 sq. km (18.05%) of the study region is made up of dense grassland. Sparse grassland falls under the category of substantial land cover and makes up 887.31 sq. km (33.96%) of the research area. Land use/land cover under water bodies, barren land, agriculturally productive regions, salt-affected areas, built-up areas, and others make up approximately 8.58 sq. km (0.32%), 579.94 sq. km (22.19%), 471.69 sq. km (13.64%), 148.45 sq. km (5.68%), 58.24 sq. km (2.22%), and 102.03 sq. km (3.90%), respectively, in the Banni grassland.

### Geomorphology

Environmental management depends heavily on geomorphology or the study of landforms and the physical processes that shape them. Geomorphological units, or the physical properties of the Earth’s surface and near-surface, are crucial for hydrogeological studies, topographical analysis, and the identification of groundwater resources^84^. Because geomorphological processes are a component of a larger system of interconnected phenomena, it is important to consider the social, economic, and cultural context of the area when evaluating their environmental importance^63^. The geographical region’s geomorphology includes high-level mudflats, which cover approximately 344.23 sq. km (13.17%) of the land; low-level mudflats with saline depressions, which cover approximately 583.47 sq. km (22.33%); lineaments; and shallow gullies, as well as mid-level mudflats, which cover approximately 1685.14 sq. km (64.49%) of the area (Fig. 6F).

### Slope

**Figure 8:**
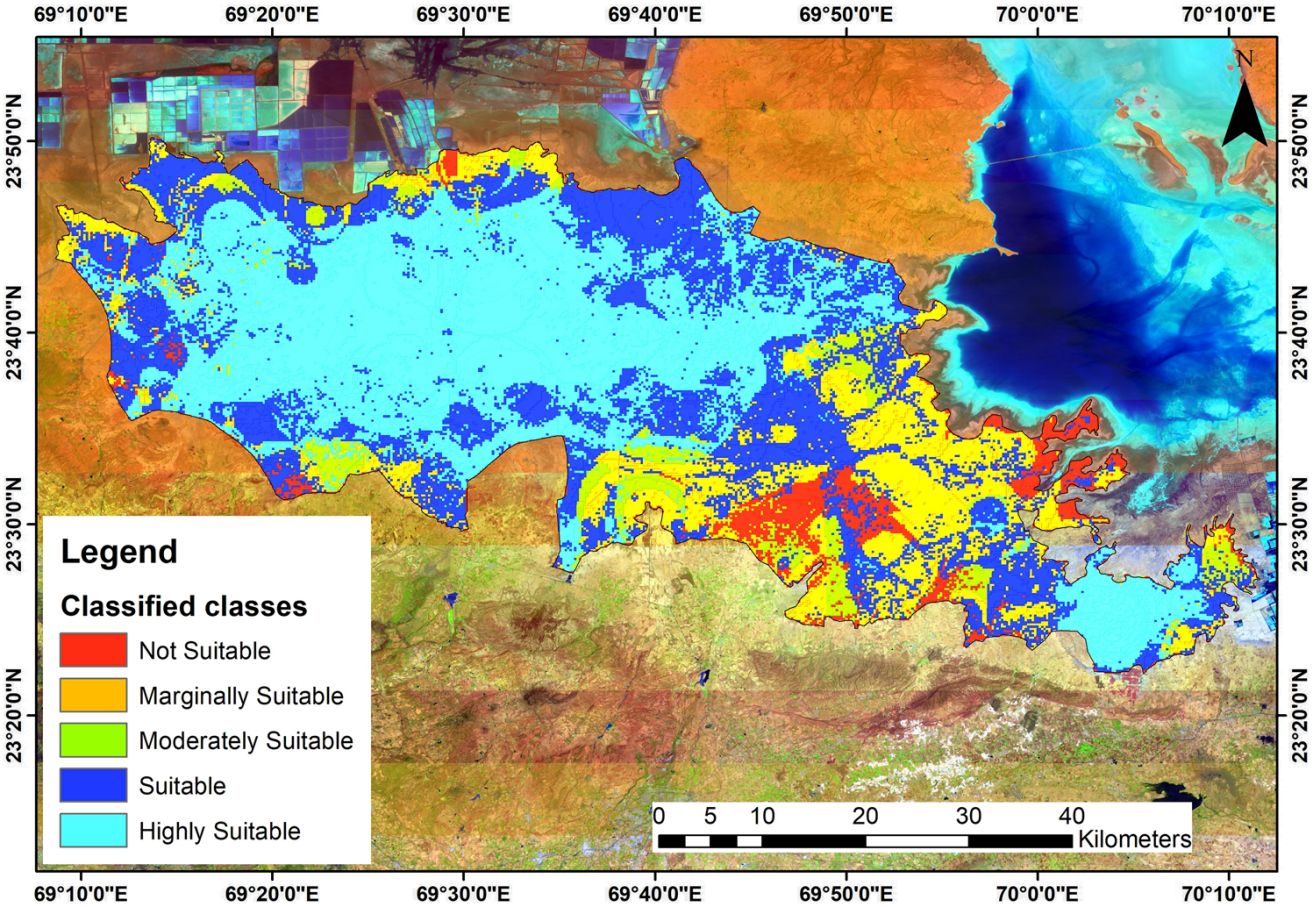
Grassland Suitability Map. The map prepared by authors using Arc GIS version 10.7 (https://desktop.arcgis.com/en/quick-start-guides/10.7/arcgis-engine-developer-kit-and-engine-quick-start-guide.htm).

In land management studies, the slope characteristics of a place are particularly important^63^. This classification assists in highlighting the primary limiting criteria for certain applications, such as plantations. Gradient limitations are particularly significant in transportation and agriculture. Because of stochastic local extinction and colonisation events, successional change or a response to changing management, the species composition of fragmented semi natural grasslands may change over time. The influences of topography (slope and aspect) on soils and the microclimate may mediate vegetation resilience to change^85^. The Banni grassland’s slope map is shown in Fig. 8. The slope values were classed and divided into flat (0–3°), gentle (3°–5°), and steep (> 5°) classes (Fig. 6G). A high weight is assigned for flat and gentle slopes. A low weight is assigned for steep slopes. Approximately 2518.41 sq. km. of the region are covered by flat land, 73.38 sq. km. by gentle slopes, and just 6.94 sq. km. by steep slopes.

### Bulk density

Soil bulk density is a fundamental yet important physical soil characteristic related to soil porosity, soil moisture, and hydraulic conductivity for the purpose of evaluating soil quality and controlling land use. Because soil bulk density varies with spatial scale and layer, complex processes of soil formation, ecological conditions, and human activities all contribute to the geographical variety of soil bulk density. Thus, knowledge of the factors influencing soil bulk density and geographical distribution can be helpful in predicting relevant soil processes and enhancing soil quality^86^. The concentration of organic matter, soil management practices, and soil particle sizes all affect the optimal and critical limits of soil BD. A bulk density of less than or equal to 1.3 g cm^−3^ is good, that between 1.3 and 1.55 g cm^−3^ is fair, and that greater than 1.8 g cm^−3^ is considered extremely poor^87^. The bulk density ranged from 1.21 to 1.83, with the majority falling between 1.21 and 1.41 and covering 1188.48 sq. km (or 45.5%) of the area (Fig. 6H). A total of 944.67 sq. km (36.1%) are in the 1.41 to 1.62 range, whereas 478.88 sq. km (18.3%) are in the 1.62 to 1.83 range.

### CEC

The cation exchange capacity (CEC) is a critical parameter for assessing the fertility and nutrient-holding capacity of soil. It measures the soil’s ability to retain and exchange cations, such as potassium, calcium, magnesium, and other essential nutrients, with the soil solution. A high CEC indicates that the soil can hold more nutrients, reducing the risk of leaching and nutrient loss^88^. By understanding the CEC of soil, farmers and agronomists can effectively manage fertiliser application, tailor nutrient management strategies, and maintain optimal soil fertility for healthy plant growth and sustainable agricultural practices. The CEC ranged from 17.26 to 145.69 meq/100 g (Fig. 6I), with the majority ranging between 17.26 and 60.07 meq/100 g and covering 1815.07 sq. km (or 69.4%) of the land. A total of 743.74 sq. km (28.4%) have values ranging from 60.07 to 102.88 meq/100 g, whereas 53.76 sq. km (20.05%) have values ranging from 102.88 to 145.69 meq/100 g.

### SAR

When assessing a soil’s suitability for agricultural uses, particularly irrigation, the SAR of the soil must be taken into consideration. The SAR calculates the soil sodium content in relation to other cations (such as calcium and magnesium). High SAR values suggest the possibility of soil sodicity, which can result in poor soil structure, decreased water infiltration, and impaired plant development. Farmers may prevent soil deterioration, increase crop yield, and improve water quality by routinely measuring SAR. A suitable agricultural soil produces SAR values lower than 13^89^. The SAR ranged from 0.01 to 1.63; however, the majority ranged between 0.01 and 0.55 and covered 1423.43 sq. km (or 54.4%) of the land (Fig. 7A). A total of 348.02 sq. km (13.32%) and 840.51 sq. km (32.1%) are in the range of 0.55 to 1.09 and 1.09 to 163, respectively.

### Grassland suitability analysis (GSA)

A combined suitability map was generated via weighted overlay analysis utilising the criterion layers with their respective weights. The available N, available P, available K, Fe, Mn, Zn, Cu, pH, SOC, soil texture, EC, rainfall, WHC, infiltration rate, LULC, geomorphology, slope, bulk density, CEC and SAR thematic reclassified polygon maps were then superimposed on this suitability map to determine the final suitability map. According to this map (Fig. 8), 35.71% (937 sq. km) of the study area is highly suitable, 27.77% (728 sq. km) is suitable, 27.23% (714 sq. km) is moderately suitable, and 6.94% (182 sq. km) is marginally suitable. Approximately 2.34% (61 sq. km) is determined to be ‘not suitable’ at all. Table 6 presents the details of the area and land characteristics of the different suitability categories for Banni grassland.

**Table 6:** Land suitability classes and their characteristics.

GSA-level	Areal extent	Land characteristics	Remarks
Highly suitable	937 sq. km	Flat to gentle slopes (0°–3°), pH low to moderate, High nutrient soils, medium to high SOC, Low bulk density and high WHC, low SAR	Land that is highly suitable for grassland restoration, If irrigation provided, dense grassland is conceivable or rainwater harvesting structures are created
suitable	728 sq. km	Generally, gentle slopes (< 5°), pH low to moderate, adequate nutrient soils, medium SOC, low to medium bulk density, medium to high WHC, low to medium SAR	Land that is suitable for grassland restoration, If irrigation and water bodies are provided, dense grassland is conceivable with conservation strategies and land management
Moderately suitable	714 sq. km	Generally, gentle slopes (< 5°), pH moderate to high, Fair soil nutrients, medium to low SOC, low to mostly medium bulk density, medium to high WHC, low to medium SAR	Land that is suitable for grassland productivity when managed properly. Soil parameters through interventions
Marginally suitable	182 sq. km	Generally, gentle slopes (< 5°), pH moderate to high, Fair to low soil nutrients, low SOC, low to mostly medium bulk density, medium to high WHC, low to medium SAR	Medium favourable for the development of grasslands under careful land management. Although it is feasible, protecting the soil from severe erosion and salt intrusion is necessary through interventions
Not suitable	61 sq. km	Generally, gentle slopes (< 5°), pH high to very high, low soil nutrients, low to very low SOC, low bulk density, high to very high EC, medium to high SAR, low to medium WHC	Grasslands and planting is not possible on these regions due to geological and environmental controls

### Validation of the grassland suitability map

The dense grassland was shown to be related to a flat slope, good soil quality, and the availability of major and minor nutrients, whereas the sparse grassland was found to be associated with a gentle slope, moderate soil quality, and low to moderate levels of soil nutrients. Moreover, low to moderate soil characteristics and soil nutrients were also connected with barren land and other LULC classifications. The validation factors of grassland suitability and development were therefore based on the thematic layers of the examined region as determined by a field study. By comparing the classified data (output) with the data from the training set, the accuracy was evaluated. Using cross-tabulation of classified data against reference data, an error matrix was obtained. When classified data are compared to reference data, their accuracy is evaluated. The cross-tabulation of categorised data against reference data yields an error matrix. The accuracy of users, producers, and overall accuracy were determined using an error matrix. In addition to determining accuracy levels, the accuracy assessment technique was also used to improve accuracy levels. Ground reference locations were chosen from the research area using the GPS technique, and they were validated using classified data. Through the use of a GPS survey, 168 ground reference points from the research region were confirmed, and they were then compared with classified data. The overall accuracy of the classified map was calculated to be 85.22% (Table 7).

**Table 7:** The error matrix for grassland suitability and ground data.

Reference data							
Classified data	Highly suitable	Suitable	Moderately suitable	Marginally suitable	Not suitable	Total samples	Users accuracy (%)
Highly suitable	30	4	1	0	0	35	89.09
Suitable	1	24	4	0	0	29	82.33
Moderately suitable	0	5	38	4	0	47	80.85
Marginally suitable	0	0	1	42	1	44	81.20
Not suitable	0	0	0	0	12	12	95.34
Total samples	32	33	44	46	13	167	
Producer’s accuracy (%)	92.50	82.51	84.67	75.63	88.12	Overall accuracy	85.22

## Conclusion

The aim of the current study was to determine the grassland development zones in the flat, undulating Banni grassland in western India by combining soil sampling analysis and remote sensing and GIS techniques. In this study, the delineation of grassland restoration zones was performed using a total of 20 thematic layers, including available nitrogen, available phosphorus, available potassium, Fe, Mn, Zn, Cu, pH, soil texture, soil organic carbon, EC, rainfall, water holding capacity, infiltration rate, land use land cover, geomorphology, slope, bulk density, sodium adsorption ratio, and cation exchange capacity. To generate land suitability analysis maps for grassland suitability, the MCDM tool with GIS was utilised. The impact of certain criteria was determined by expert analysis and views, which were then estimated using MCDM and the AHP, which was used to assign weights. According to our findings on grassland patterns and suitability, highly suitable land (937 sq. km) and suitable land (728 sq. km) have no significant restrictions if water sources are provided either through irrigation or rainwater harvesting. Grassland expansion is also feasible in somewhat appropriate areas (moderately suitable—714 sq. km), although careful land management is needed. For grassland development, there is marginally suitable land (182 sq. km) and not suitable land (61 sq. km), necessitating terracing, supplementary inputs such as fertilisers, protection from high runoff and erosion, salt intrusion, etc. As a consequence, decisions may be made knowing exactly how they will affect future land use development and how they will affect the impacts.

Therefore, it is essential to assess grassland degradation thoroughly along with climate, topography, vegetation, and soil characteristics and use various techniques for analysis, such as remote sensing, to systematically analyse the suitability of grassland development, thereby improving the science and accuracy of the research. Since degraded grasslands make up the majority of the study region, this research will enhance the area’s grassland productivity and fertility database with the aid of required inputs. The Banni grassland of western India was once considered the finest grassland of Asia; however, owing to various natural and anthropogenic factors, it is now on the verge of drastic degradation. In this work, we incorporated edaphic and proximal GIS-based factors to provide a concise view, taking into consideration all necessary factors required for the sustenance of a grassland ecosystem. The Banni grassland is an ecosystem for which ecological vulnerabilities need to be considered while considering geo-ecological perspectives. The findings of this study have significant implications for the conservation and sustainable use of grasslands in western India. By identifying areas with high ecological value and suitability, this research provides valuable guidance for policymakers, land managers, and local communities in making informed decisions regarding land management practices. The findings can help generate evidence-based recommendations for sustainable grassland management, including biodiversity conservation and the enhancement of livelihoods for local communities dependent on grasses for their livestock. Furthermore, the study's methodology and outcomes can be replicated and applied to similar grassland ecosystems in other regions. This research contributes to the broader understanding of grassland management and restoration practices, enabling the transfer of knowledge and best practices to other areas facing similar ecological challenges. Overall, this study underscores the importance of assessing land suitability for sustainable grassland management and highlights the potential for maximising the ecological value of grasslands in western India and beyond. By integrating scientific data, stakeholder consultations, and expert knowledge, this research offers a holistic approach to land management, fostering ecological restoration and promoting the long-term sustainability of grassland ecosystems.

## Data Availability

The datasets generated during and/or analysed during the current study are available from the corresponding author upon reasonable request.
